# Wavelength-specific optoacoustic-induced vibrations of the guinea pig tympanic membrane

**DOI:** 10.1117/1.JBO.26.3.038001

**Published:** 2021-03-05

**Authors:** Larissa Heimann, Christopher Carlein, Katharina Sorg, Rolf Diller, Achim Langenbucher, Bernhard Schick, Gentiana Ioana Wenzel

**Affiliations:** a Saarland University, Medical Center, Department of Otolaryngology, Homburg, Germany; b University of Kaiserslautern, Department of Physics, Kaiserslautern, Germany; c Saarland University, Medical Center, Department of Experimental Ophthalmology, Homburg, Germany

**Keywords:** tympanic membrane, optoacoustic, visible/near infrared, laser Doppler vibrometry, auditory prostheses, hearing

## Abstract

**Significance:** Optoacoustic-induced vibrations of the hearing organ can potentially be used for a hearing device. To increase the efficiency of such a hearing device, the conversion of the light energy into vibration energy within each type of irradiated tissue has to be optimized.

**Aim:** To analyze the wavelength-dependency of optoacoustic-induced vibrations within the tympanic membrane (TM), and to define the most efficient and best-suited optical stimulation parameters for a novel auditory prosthesis.

**Approach:** Single nanosecond laser pulses, continuously tunable in a range of visible to near-infrared, were used to excite the guinea pig TM. The induced vibrations of the hearing organ were recorded at the malleus using a laser Doppler vibrometer.

**Results:** Our results indicate a strong wavelength-dependency of the vibration’s amplitude correlating with the superposition of the absorption spectra of the different specific tissue components.

**Conclusions:** We investigated the spectrum of the vibrations of the hearing organ that were induced optoacoustically within a biological membrane embedded in air, in an animal model. First applications for these results can be envisioned for the optical stimulation of the peripheral hearing organ as well as for research purposes.

## 1 Introduction

The optoacoustic effect is widely used in medicine and industry for photoacoustic spectroscopy and photoacoustic imaging, e.g., tomography or microscopy.^1^^,^^2^ This effect is the result of the absorption of pulsed light in an absorber medium inducing a thermal expansion and contraction of the substrate. Through these periodic variations of the tissue density, a sound source is created. Previous reports demonstrate that this effect could potentially be used to stimulate the hearing organ to compensate for deficits in patients who are not sufficiently supplied with currently available hearing aids.^3^^–^^5^ A detailed optoacoustic stimulation strategy to vibrate the hearing organ using a custom-designed laser pulse amplitude modulation is described by Stahn et al.^6^

In the hearing system, sound waves travel through the ear canal and induce vibrations of the eardrum. These waves are transferred through the ossicular chain, located within the middle ear further to the inner ear, the cochlea. Here, this mechanical information is transcribed in electrical impulses, a process known as mechanoelectrical transduction, and transferred through the auditory nerve to the central nervous system. Laser pulses delivered to the hearing organ lead to vibrations of the irradiated vibratory structure.^6^^,^^7^ These vibrations are then transmitted to the sensory cells within the inner ear through the physiological hearing pathway just described (e.g., eardrum, middle ear, and inner ear). Commercially available hearing aids^8^ are not used by some of the hard of hearing people due to discomfort in the ear canal, occlusion effect, auditory canal inflammations through the earpiece, feedback, and insufficient frequency specificity.^6^ Other auditory prostheses, such as bone-conduction hearing implants^9^ or cochlear implants,^8^ require surgery or permanent skin contact. Ultrasound stimulation,^10^ a novel technique that is still in development, would require as well direct contact to the tissue to transmit the induced vibrations. Optoacoustic stimulation could replace the speaker of the currently available hearing aids with a specially designed, non-occluding light source to activate the hearing organ without the need for direct contact with the vibratory structure. To optimize the optoacoustic stimulation for biocompatibility and energy efficiency reasons, we sought to determine the best-suited wavelengths for the stimulation at the eardrum level, as one of the first and most accessible structure of the peripheral hearing organ.

In 2009, Zhang et al.^7^ demonstrated the optoacoustic-induced vibrations within explanted inner ears, with two wavelengths (355 and 532 nm). Later Schultz et al.^4^ presented the stimulation of the inner ear as well by ns-laser pulses at several wavelengths. He worked with anesthetized guinea pigs using compound action potentials (CAPs) as a recording method. They concluded that the stimulation with visible (VIS) and near-infrared (IR) light based on the optoacoustic effect at the inner ear level involves water and hemoglobin as the main absorbing components. However, the inner ear is a fluid-filled organ and the question remains, which are the optimal stimulation parameters in other parts of the auditory pathway, not embedded in fluid, e.g., the eardrum. In contrast, the eardrum is surrounded by air and differs in structure, composition, and vibration characteristics compared to the inner ear,^11^ facts that are not allowing a complete transfer of the data recorded by Zhang et al.^7^ and Schultz et al.^4^

The purpose of this study was to determine the best-suited wavelengths for a light-based stimulation of air-embedded structures of the peripheral hearing organ. This would allow us to customize and optimize the optoacoustic stimulation depending on the patient’s still functional vibratory structures.^6^^,^^12^

## 2 Materials and Methods

We opted to analyze the spectrum of the optoacoustic-induced vibrations at the eardrum level using a laser Doppler vibrometer (LDV) *in vitro*.

### 2.1 Animal Model

**Fig. 1:**
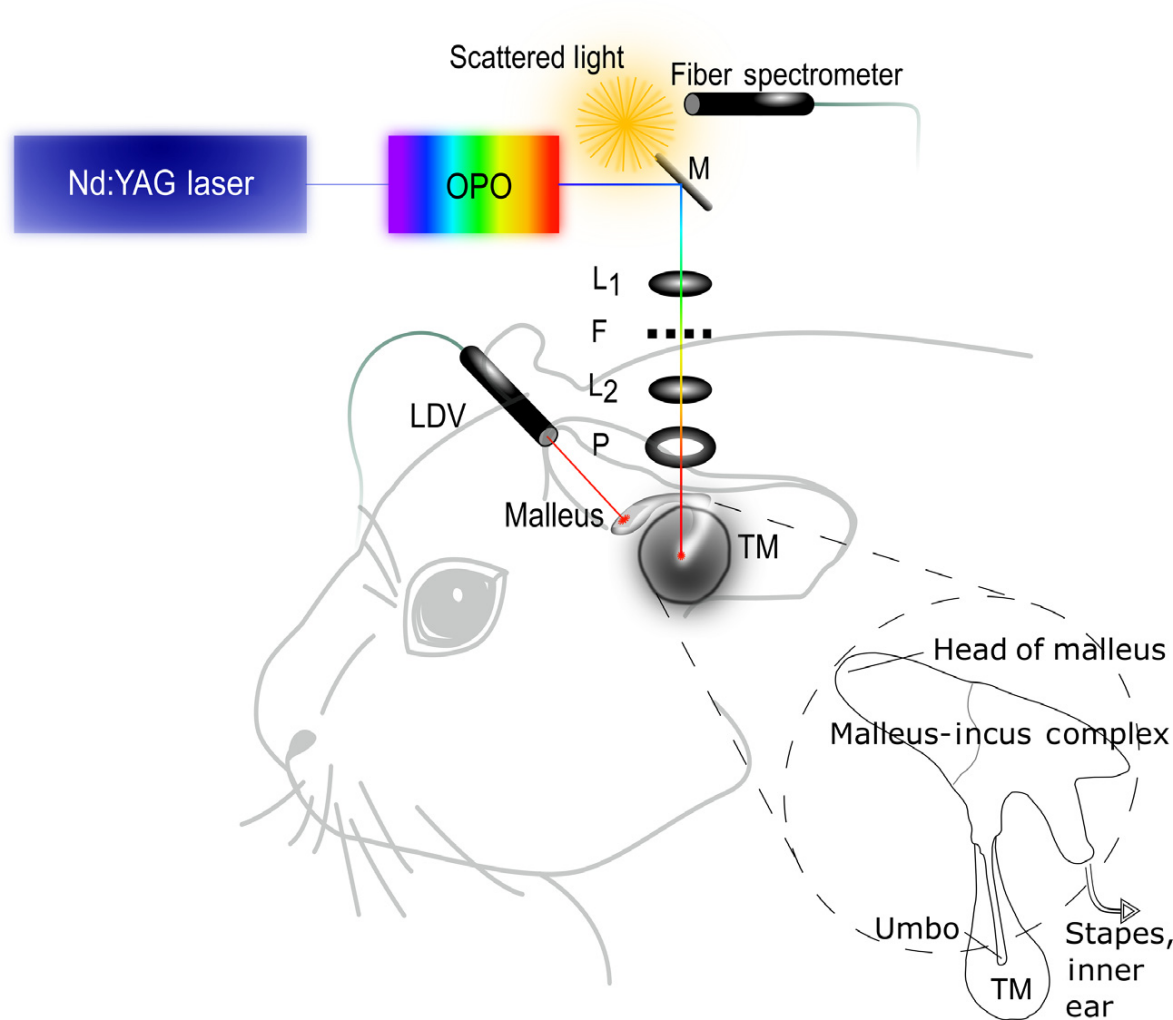
Experimental setup. The wavelength of a frequency-tripled Nd:YAG laser was modulated by an OPO system. The stimulating laser beam was focused on the umbo, the central part of the TM, using two lenses (L1, L2). A pinhole (P) reducing scattered light and a spectral filter (F) were also placed within the optical path. A movable mirror (M) was used for adjustments. The wavelengths were measured by an absorption spectrometer (fiber spectrometer). An LDV was positioned on top of a retroreflector attached to the head of the malleus. The schematic of the malleus-incus complex is drawn after Mason.^15^

Female albino guinea pigs (Charles River Laboratories, Solingen, Germany) weighing 450 to 650 g were anesthetized with 40  mg/kg ketamine (Ketanest, Albrecht, Aulendorf/Württemberg, Germany) and 10  mg/kg xylazine per kg body weight (Rompun, Bayer Health Care, Leverkusen, Germany), applied intramuscularly. They were then euthanized in deep anesthesia following the protocols approved by the Central Veterinary Office of Saarland in accordance with the German Animal Welfare Law. The animals were decapitated and a metal rod was fixed on the frontal bone with screws and additionally secured with cement to be used for the fixation of the head during recordings. The outer ear was dissected, the cartilaginous outer ear canal was removed, and the tympanic membrane (TM) was exposed. The dissected skulls were then stored frozen at −18°C until the planned measurements. For the LDV recordings, the samples were defrosted at room temperature for a minimum of one hour, being covered by a wet paper tissue to maintain and control the humidity of the sample. A comparable humidity between the samples was mandatory since it is one of the parameters, which could affect the absorption of photons within the tissue and therefore the efficiency of the optoacoustic effect. The TM and the head of the malleus, the first ossicle of the middle ear, were exposed. The manubrium of the malleus is directly attached to the TM, forming a concavity with its deepest part located in the middle of the eardrum, the umbo.^13^^,^^14^ We used 11 ears to establish and standardize the technique and 5 ears for the herein presented experiments. Each sample studied was fixed in position and the laser light was then positioned at the umbo (Fig. 1). The induced vibrations could be detected by an LDV (Polytec, Germany, OFV 501/2602; translation: 5  mm/s per V) measuring the vibration velocity (cf. Sec. 2.3).

### 2.2 Laser Stimulation

We used a frequency-tripled Nd:YAG laser (Quanta-Ray-INDI, Spectra-Physics) light source emitting pulses with an average energy of 80±4  mJ at 355 nm (pump), and pulse duration of 50 ns measured at full width at half maximum (FWHM) at a repetition rate of 11 Hz (Fig. 1).

The wavelength of the laser pulses was varied by an optic parametric oscillator (OPO) (GWU, versaScan, Germany) in a range of 420 and 2200 nm. The laser beam was attenuated by neutral density filters to a very low intensity during the visual adjustment for safety reasons. Two convex lenses were used to first collimate the laser beam and then to focus it on the TM. To ensure the accuracy of the irradiation location, the spot was positioned on the umbo via an adjustable mirror under visual control. The irradiated area was $0.2\;{\mathrm{mm}}^{2}$, allowing the stimulation of a representative average composition of the TM to induce the optoacoustic effect. For monitoring, a camera was used to make the IR light VIS.

Nonlinear phenomena inside the OPO system caused the splitting of the pump laser pulses into the signal pulses (high-energy photons) and idler pulses (low-energy photons), obeying the law of conservation of photon energy. For tuning the wavelength, the angle between the pump laser beam and the beta barium borate crystal of the OPO was adjusted. The idler was used for the recordings of vibrations induced by 800- to 2200-nm pulses and the signal with higher photon energy levels, for the pulses between 400 and 700 nm. A spectral bandwidth (FWHM) of 5 nm was determined for the used signal pulse at 420 nm. The wavelength of the signal pulses was measured with an optical absorption spectrometer (Ocean Optics, USB2000+), whereas the wavelength of the idler pulses was calculated using the wavelength of the signal and the pump pulses.

For each wavelength, 128 oscilloscope triggered sweeps per measurement were averaged at a sampling rate of 0.5 to 10 MHz (Table S1 in the Supplementary Materials). The measurement was repeated three times. The LDV and the laser operated continuously; therefore, the experimental setup did not need an external trigger. If the signal level was too low to be sensed by the oscilloscope as triggered sweeps, the oscilloscope’s free-running mode was used and the signals were manually detected. To spectrally separate the signal and idler, two complementary filters were used as a bandpass for the signal and the idler pulses, respectively.

**Fig. 2:**
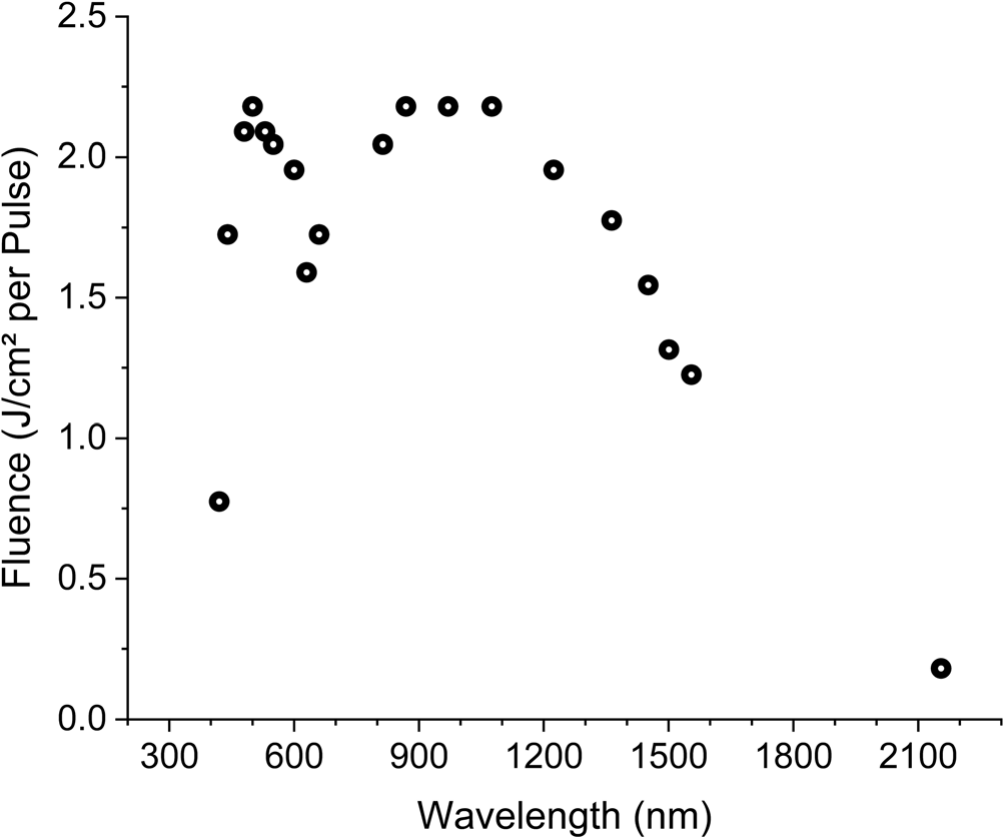
Laser fluence spectrum.

The vibration velocity (cf. Sec. 2.3) varied linearly with the laser power within the used laser power level. This proportionality allowed normalization of the vibration velocity with respect to the laser power P(λ) (cf. Fig. 2). Equation (1) was used to calculate the effective velocity $v_{\mathrm{cor}}(\lambda )$, which then was normalized to the maximum value of $v_{\mathrm{cor}}$. The error $\Delta v_{\mathrm{cor}}$ [Eq. (2)] was calculated using error propagation with the standard deviation σ(v) for the error of v(λ) and the measured error ΔP=±3  mW.

$$v_{\mathrm{cor}}(\lambda )=\frac{v(\lambda )}{P(\lambda )},$$ (1)

$$\Delta v_{\mathrm{cor}}=|\frac{\partial v_{\mathrm{cor}}}{\partial v}|\sigma (v)+|\frac{\partial v_{\mathrm{cor}}}{\partial P}|\Delta P=\frac{\sigma (v)}{P(\lambda )}+\frac{\Delta Pv_{\mathrm{cor}}}{P^{2}(\lambda )}.$$ (2)

The calculation of the frequency spectrum of v(λ) has been performed using the windowed Fourier transform tool of the program Origin(Pro) 2020.^16^

The velocity of the measurements with 800- to 2200–nm pulses was in the range of the LDV resolution limit. The signal-to-noise ratio depended on the experimental conditions [laser fluence (Fig. 2), humidity, tissue’s blood concentration, exact focus position, and the size of the hearing organ, etc.] leading to a different number of the recorded data points for each ear within the analyzed spectrum (Fig. 4).

### 2.3 Vibration Measurements

This method was inspired by the work of Zhang et al.^7^ and adapted to our experimental setup. Using a red, single point LDV, the recording LDV laser beam was first visually focused on a commercial retroreflector foil piece ($∼0.5\;mm^{2}$) fixed on top of the head of the malleus making sure that no other bony structures around the opening had contact with it. The focus was then manually optimized using a build-in lens at the end of the LDV laser fiber. The LDV signal level was monitored over the LDV display signaling LED and used to align the laser beam perpendicular to the reflector surface. The laser fiber was mounted on a holder and fixed in its optimal position. The vibration velocity of the head of the malleus was then recorded using LDV.^17^^,^^18^ The LDV measuring principle is based on interferometry detecting the displacement of a moving surface over time. Its velocity parallel to the LDV laser beam is tracked.

Before starting the recordings, an acoustic test signal was applied to the eardrum to optimize the LDV signal level. Due to the variable signal level of the recordings for 420  nm≤λ≤650  nm (far away from the resolution limit), the vibration velocity was recorded in steps of 10 to 50 nm. For λ>800  nm (in the range of the resolution limit), the wavelength was tuned continuously and data were recorded at all wavelengths for which an LDV signal was detectable.

## 3 Results

**Fig. 3:**
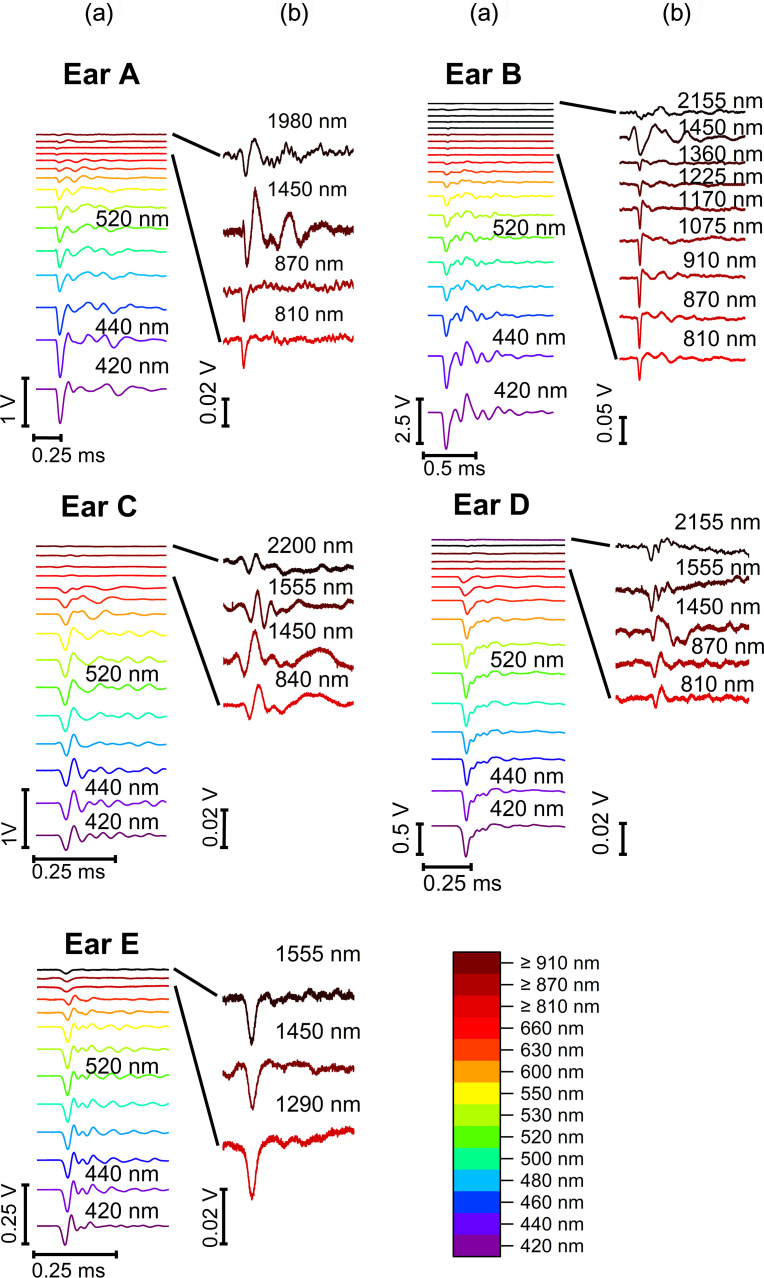
Raw data of the vibrations induced by optoacoustic stimulation at the TM (umbo) in guinea pigs (5 ears, A to E), recorded with an LDV at the head of the malleus. Plotted is the velocity v(t) of the optoacoustic-induced vibrations after optical excitation at different wavelengths. (a) All ears from bottom to top: 420, 440, 460, 480, 500, 520, 530, 550, 600, and 630 nm; ear A: 660, 810, 870, 1450, and 1980 nm; ear B: 660, 810, 870, 970, 1075, 1170, 1225, 1360, 1450, and 2155 nm; ear C: 840, 1450, 1555, and 2200 nm; ear D: 660, 810, 870, 1450, 1555, and 2155 nm; ear E: 1290, 1450, and 1555 nm. (b) Enlarged measurements at the displayed wavelengths.

The vibration velocity measured at the head of the malleus while optoacoustically stimulating the TM at different wavelengths (420 to 2200 nm) demonstrated the highest vibration amplitude at the beginning of each recording. As a result of the tissue damping, the vibration velocity decreased to lower amplitudes over all our recordings (Fig. 3).

### 3.1 Wavelength-Dependent Distribution of the Optoacoustic-Induced Vibrations

**Fig. 4:**
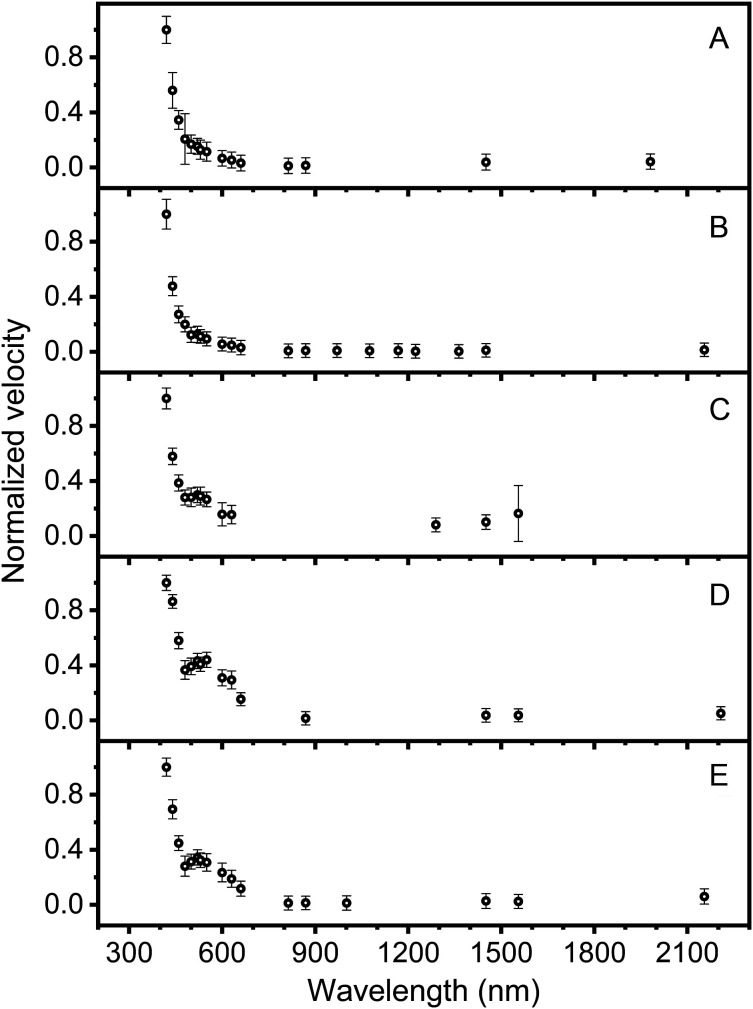
Vibrations induced by optoacoustic stimulation at the TM (umbo) in guinea pigs (5 ears, A to E), recorded with an LDV at the head of the malleus. Plotted is the effective velocity $v_{\mathrm{cor}}(\lambda )\pm \Delta v_{\mathrm{cor}}$ of the optoacoustic-induced vibrations after optical excitation at different wavelengths, normalized to the maximum value within each ear.

As expected, the data demonstrated a variation of the vibration velocity depending on the applied wavelength (Fig. 4). The highest vibration velocity of 100 % was induced at 420 nm. A further maximum could be identified between 520 and 550 nm demonstrating variations of its position and amplitude between the individual ears (n=5). The irradiated ears could be parted into two groups (Fig. 4): A and B with a very weak maximum and C to E with a pronounced maximum between 520 and 550 nm. The vibration value induced in the near-IR (idler pulses) demonstrated on average a 93 % lower signal level compared to the vibrations evoked with UV/VIS light (signal). The value of the optoacoustic signal induced at 1450 nm was present in the recordings of all ears. Within another set of recordings (ear B, Fig. 4) in which data points representing the TM vibrations induced through laser pulses with wavelengths between 810 and 1450 nm, a very low signal level could be observed. A trend can be suggested for another vibration maximum around 1170 nm and two further peaks at 870 and 1000 nm. The measured amplitude of the velocity for ear B was above the resolution limit of the LDV and therefore well detectable (see Sec. 4 for further discussion). Additionally, another peak could be observed at 1555 nm within this second group (ear C to E, Fig. 4). Above 1370 nm, the vibration velocity increased slightly with increasing wavelength in all ears. The error of the peak position depended on the step size of the measurements and was at least±10  nm.

**Fig. 5:**
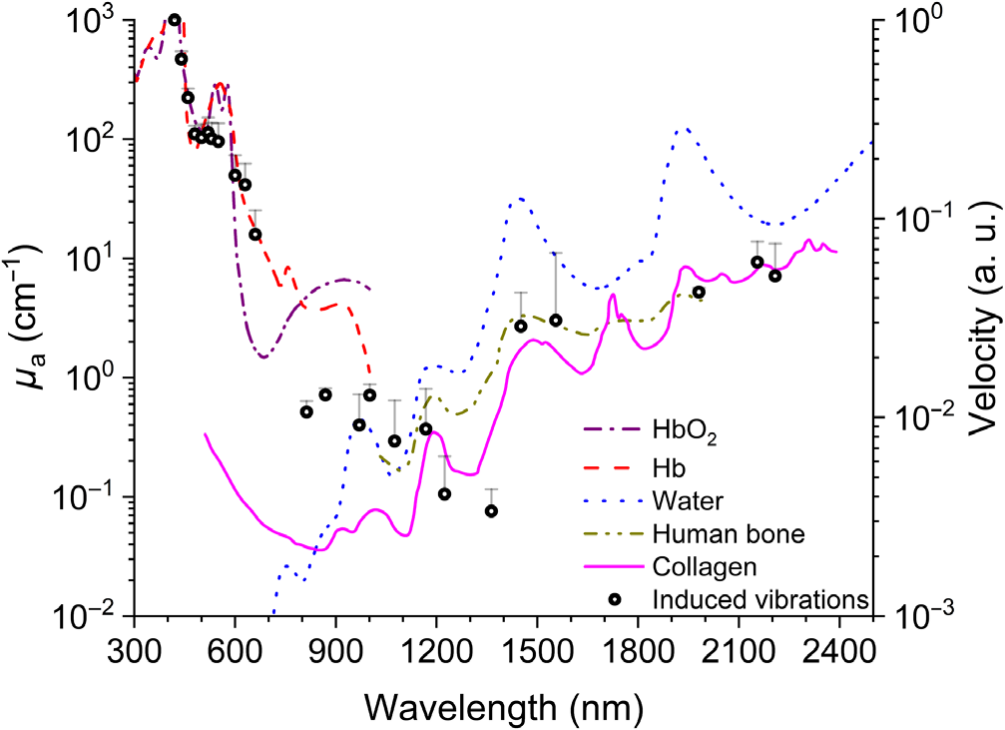
The velocity of the vibrations induced by the optoacoustic effect correlated to the absorption coefficient $\mu_{a}$ of hemoglobin (Hb) and oxyhemoglobin (${\mathrm{HbO}}_{2}$),^19^ water,^20^ collagen,^21^^,^^22^ and human cranial bone.^23^ Error bars are shown as the standard error of the mean. For reasons of clarity and comprehensibility, negative error bars are not displayed.

An overview of the averaged wavelength-dependent measurements of the optoacoustic-induced vibrations (black circles) is presented in Fig. 5. Different absorption spectra from literature are inserted in colored lines into the diagram to visualize the significance of the individual progression recorded in our experiments. To average the data of all ears, the mean and standard error of the mean for each wavelength were calculated. If a measurement point could only be recorded in one ear, no mean value was included. The averaged data present a good correlation to the absorption spectrum of hemoglobin and oxyhemoglobin^19^ between 400 and 700 nm. Further, between 800 and 2300 nm, the induced vibrations reveal first a decreasing signal, then a step-like shift toward higher and further increasing values at around 1400 nm. Furthermore, the data fluctuation correlated with the absorption spectra of water,^20^ collagen,^21^^,^^22^ and human cranial bone.^23^

### 3.2 Differences in Vibration Properties between Ears

**Fig. 6:**
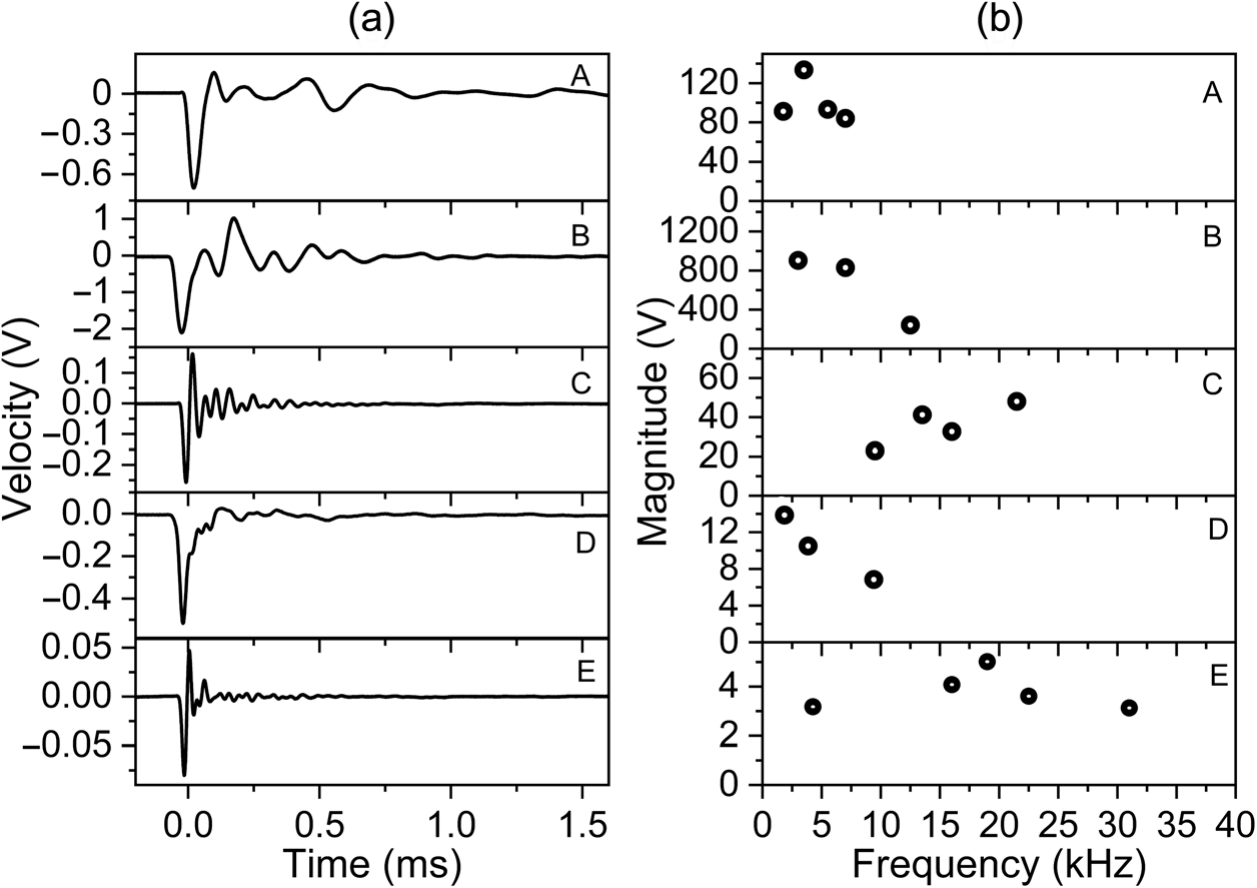
(a) Laser pulse-induced vibrations at 420 nm and (b) frequency peak analysis of the five different ears A to E.

We divided the five ears into two groups concerning the temporal evolution of the recorded vibrations and their calculated frequency spectrum. Depending on the LDV signal in the time domain (Fig. 6), the vibrations of group 1 (A and B) demonstrated higher damping (1 ms until the signal disappeared) as compared to group 2 (C to E), consisting of frequencies between 2 and 21 kHz with a maximum frequency around 3 kHz. The second group exhibited half of the decay time (0.5 ms) and frequencies larger than 21 kHz, with the highest magnitude around 20 kHz. An exception was ear D displaying the maximum represented frequency at 2 kHz. Generally, each ear exhibited its vibrational pattern with unique frequency characteristics and frequency maxima at 3.5, 3, 21.5, 1.5, and 19 kHz for sample A to E, respectively.

## 4 Discussion

Commercially available hearing aids are based on the amplification of the incoming sound. The optoacoustic-based hearing device that we propose to develop, as described by Stahn et al.,^6^ translates the incoming acoustic signal into modulated laser pulses. Therefore, instead of transmitting the amplified sound to the ear, the vibration of the hearing organ would be induced through optoacoustic stimulation. The experiments by Stahn et al. achieved frequency-specific activation of the auditory system by a convolution-based laser pulse modulation. For the potential use of this technique in a hearing aid, further stimulation parameters, e.g., the optimal laser wavelength, have to be defined as well as additional research for the optimization of the stimulation method has to be performed.

To investigate and evaluate the optoacoustic effect on the ear, three stages of the activation process have to be considered:

1. The induced ultrasound vibrations at the stimulation location (here the TM, Fig. 1) can be recorded to detect the photoacoustic signal. This method is used in photoacoustic spectroscopy and photoacoustic imaging mostly with air conducted ultrasound transducer instead of LDV due to better signal-to–noise ratio.^24^
2. The middle and/or inner ear mechanical vibrations induced by these ultrasound waves within the peripheral auditory organ in a distance from the stimulation focus can be recorded using the LDV laser beam positioned at a vibratory structure of the hearing organ (here the head of the malleus, Fig. 1).
3. The neuronal activity *in vivo* that includes the neuronal processing of the signal. The detection of auditory brainstem responses^25^ or neural recordings in the central nucleus of the inferior colliculus^6^ are examples of such experimental setups.

Depending on the investigated structure and experimental aim, 1D, 2D, or 3D LDV recordings can reveal a very good insight into TM vibration pattern^26^ or middle ear vibration behavior. The goal of the herein presented experiments was to assess from the mechanical point of view, which wavelength is most suitable for a hearing device. We, therefore, chose *in vitro* experiments to reduce the number of variables induced by neuronal activity. For these experiments, a single point LDV was sufficient to determine the amplitude of the vibration velocity at the middle ear ossicles and characterize the vibration spectrum (Fig. 5).

In this study, the optoacoustic-induced vibrations of the hearing organ in an animal model indicate a strong wavelength-dependency of their amplitude correlating with the absorption spectra of the different tissue components.^27^

A laser pulse absorbed by the tissue and matching the parameter for stress confinement causes a change in tissue density and pressure.^28^^,^^29^ As a consequence, wideband ultrasound waves travel through the tissue presenting unique frequency maxima for each tissue type.^30^ These ultrasound waves induce vibrations of the entire hearing organ at frequencies within the audible spectrum.^10^^,^^31^ The conversion of the ultrasound to perceptual sound is not well understood, yet. Computer-based simulation of these coupled vibration systems is expected to give further insight into this phenomenon.^32^

The optoacoustic-induced sound waves occur at every tissue component that absorbs the laser pulse. This creates multiple sound sources, e.g., at the TM surface, blood vessels, or the bony ossicle (malleus), which interfere and cause a specific vibration pattern [Fig. 6(a)]. However, the light within the optical windows (650 to 950, 1100 to 1350, and 1600 to 1870 nm)^33^ penetrates deep into the tissue. Our results demonstrated a weak or non-detectable optoacoustic signal within the optical windows of our used spectrum as well. In these cases, it can be assumed that multiple, weak sound sources along the penetrating beam are created with amplitudes beyond the detection threshold.

Additionally, as mentioned earlier, the composition and structure of the TM are critical features for the stimulation. It is a layered membrane, with an inner mucosal layer, an outer epidermal layer, and an intermediate layer of radial and circular collagen fibers as its major structure. The relative composition and mass distribution of these layers vary throughout the TM.^34^ Even though estimations regarding the compositions of the TM of the guinea pig are difficult, because of the little knowledge in the literature and the inhomogeneous behavior, it is fair to say that the inner mucosal and outer epidermal parts are relatively thin compared to the fibrous intermediate layer.^35^^,^^36^ Since the thick middle lamina contains mostly collagen fibers and the TM additionally houses blood vessels and nerves that supply it, collagen and hemoglobin are together with water the main absorption components in the TM.^36^ The overall thickness of the TM is inconstant.^34^ The thin TM at the umbo allows at least part of the laser pulses to pass through and excite the malleus-incus complex, which hence is another absorption structure.

To get a better insight into the overall process of the optoacoustic-induced vibrations in such a complex structure, we analyzed the correlation between the observed optoacoustic spectrum and the absorption coefficients of the main tissue components (Fig. 5). The measured vibration spectrum demonstrated a good correlation to the absorption coefficient of hemoglobin, oxyhemoglobin, water, bone, and collagen. The region between 420 and 800 nm displayed similar peaks as the absorption spectrum of hemoglobin (blood concentration).^19^ The prominent peak around 555 nm especially is matching the deoxygenated and oxygenated hemoglobin. Additionally, due to the irradiation of the umbo, as a combined membranous and bony structure (the malleus behind the TM), the absorption properties of bone and collagen might dominate the optoacoustic effect in the presented recordings. In our region of interest, the maxima of the absorption coefficient of water and bone were very similar (Fig. 5).^23^ Other components of the bone apart from water and collagen, such as amino acids or hydroxyapatite, absorb outside the region of interest or nearly constantly stable. ^37^^,^^38^ Vibration values around 2200 nm indicate the contribution of further absorbing components. Since collagen is a major component of the TM, it likely contributes to the measured spectrum. Park et al.^39^ measured the effective photoacoustic absorption spectrum for collagen-based tissue. They observed a step-like shift toward higher values at 1400 nm, which is suggested by our data as well (Fig. 5). However, the absorption of water is stronger having a 10 to 100 times higher absorption coefficient.

Additionally, our results at 420 to 660 nm are in line with the observations described by Schultz et al.,^4^ who recorded CAP amplitudes during cochlear stimulation. Both the shape of the optoacoustic vibrations and the CAP amplitudes follow the absorption coefficient of hemoglobin that is present within the cochlea and the TM. Differences occur in the range between 800 and 2200 nm. In this spectral range, the data of our work can be dedicated to the absorption coefficient of water but also collagen. This novel finding is in line with the comments of Schultz et al.^4^ regarding the additional contribution of unknown absorption components. Other factors adding further to the observed differences cannot be excluded either, e.g., the Grüneisen parameter.^29^^,^^40^ However, due to the inhomogeneous structure of the TM, an absolute correlation and direct calculation of its effect on the resulting optoacoustic signal as a single value would be not accurate either.

Depending on the sample with its biologically based differences, different vibrations were induced by the same applied laser pulses. The most likely explanation is the stimulation of the TM via different sound waves. The TM vibrations consist of traveling waves and standing waves.^41^ Rosowski et al.^42^ described the displacements of the TM surface in response to sound as a result of a combination of low-order modal response patterns and traveling waves. It is possible that minimal deviations of the laser spot have an impact on the type of induced sound waves of the TM and the vibrational frequency of the ossicles. The optoacoustic effect initially creates a sound source within the irradiated tissue (TM, malleus). The actual eardrum vibration deflecting the entire membrane is excited by these intrinsic vibrations. Therefore, the excited frequencies of vibrations are strongly dependent on the location of the optoacoustic sound sources. The angle between the laser beam and the TM might also contribute to this phenomenon by changing the TM components ratio excited by the laser pulses.

Another explanation for the differences in the vibration pattern could be that an unnoticed accumulation of water behind the TM increased the mass of the TM causing more dominant lower frequency vibrations of the middle ear ossicles of samples A and B. This is in line with the different vibration properties of the samples around 550 nm. Here, the maximum for water is more prominent compared to the maximum corresponding to hemoglobin, even though, no accumulated water was noticeable during sample preparation.

Schacht et al.^43^ investigated laser-induced tissue remodeling of the TM and did not observe remodeling applying laser energy of 0.3 J ($280\;J/{\mathrm{cm}}^{2}$). In our experiments, the laser energy level per pulse was kept below this value (Fig. 2) to avoid damage to the TM structure. Since in our experimental setup the laser power decreased for λ>1450  nm (Fig. 2), the resulting optoacoustic-induced vibrations were somehow beyond the detection threshold. Therefore, the wavelength was tuned continuously between 1450 and 2300 nm, and data recorded at every wavelength vibrations were induced. This explains the irregular gaps between data points (Fig. 4). With this method, we could only reveal the maxima of the optoacoustic effect for λ>1450  nm, and additional *in vivo* experiments are needed to fill up the missing data.

It is to be expected that this alternative stimulation method for hearing aids, using the light stimuli instead of sound amplification, would avoid interference between incoming and amplified soundwaves^6^ offering as well higher flexibility to the individual patients’ anatomical characteristics as well as a non-contact application strategy. The auditory prostheses should be miniaturized and designed as comfortable as possible. Therefore, the laser dimensions and power consumption should be small-sized and low. Lasers diodes currently fulfilling these conditions^44^ offer a range from UV to the near-IR spectrum. Recent research about light sources in photoacoustic imaging presented a fingertip laser diode system with potential use as a wearable device.^45^ Using a laser diode operating at a wavelength that leads to a strong optoacoustic signal and slope efficiency at the same time has the potential to be the first approach to new light-based auditory prostheses. The results of biocompatibility studies performed by Sorg et al.^25^ and Pillong et al.^46^ have to be considered for a final decision about the most suitable laser source for stimulation in new light-based auditory prostheses. Further applications for these results could include contactless induction of vibrations in various biological tissues for research purposes as well.

## 5 Conclusion

We herein investigated the spectrum of the induced vibrations of the hearing organ by optoacoustic stimulation of a biological membrane embedded in air, in an animal model. Based on our data recorded applying a laser spectrum of 420 to 2300 nm, we identified a correlation between the vibratory spectrum and the light absorption spectrum of water (420 to 2300 nm) and hemoglobin (420 to 660 nm). This is in line with the observation of Schultz et al.^4^ in their studies of the inner ear. Additionally, our investigation demonstrates that collagen can be considered as a further component contributing to the optoacoustic spectrum in the range of 800 to 2200 nm. First applications for these results can be envisioned within the optical stimulation of the peripheral hearing organ as well as for research purposes.
