# Supplementary material for: Wavelength-specific optoacoustic-induced vibrations of the guinea pig tympanic membrane
Source: J Biomed Opt. 2021 Mar 5;26(3):038001. doi: 10.1117/1.JBO.26.3.038001 (PMC7934890; doi:10.1117/1.JBO.26.3.038001)
Supplement: Supplementary file 1 [file JBO_026_038001_SD001.pdf]

Supplementary information

Tab. S1. Sample interval and sample rate of the raw data for each wavelength and all ears (A – E).

Ear	Wavelength (nm)	Sample Interval (10^{-7} s)	Sample Rate (Hz)
A	420	4.0	2,500,000
	440	4.0	2,500,000
	460	4.0	2,500,000
	480	4.0	2,500,000
	500	4.0	2,500,000
	520	4.0	2,500,000
	530	4.0	2,500,000
	550	4.0	2,500,000
	600	4.0	2,500,000
	630	4.0	2,500,000
	660	4.0	2,500,000
	813	4.0	2,500,000
	869	4.0	2,500,000
	1451	4.0	2,500,000
	1981	4.0	2,500,000
B	420	2.0	5,000,000
	440	2.0	5,000,000
	460	2.0	5,000,000
	480	2.0	5,000,000
	500	2.0	5,000,000
	520	2.0	5,000,000
	530	2.0	5,000,000
	550	2.0	5,000,000
	600	2.0	5,000,000
	630	2.0	5,000,000
	660	2.0	5,000,000
	813	2.0	5,000,000
	869	2.0	5,000,000
	970	2.0	5,000,000
	1075	2.0	5,000,000
	1168	1.0	10,000,000
	1224	1.0	10,000,000
	1363	1.0	10,000,000
	1451	2.0	5,000,000
	2155	4.0	2,500,000
C	420	2.0	5,000,000
	440	2.0	5,000,000
	460	2.0	5,000,000
	480	2.0	5,000,000
	500	2.0	5,000,000
	520	2.0	5,000,000
	530	2.0	5,000,000
	550	2.0	5,000,000

	600	2.0	5,000,000
	630	2.0	5,000,000
	660	2.0	5,000,000
	869	2.0	5,000,000
	1451	2.0	5,000,000
	1555	1.0	10,000,000
	2208	1.0	10,000,000
D	420	20	500,000
	440	20	500,000
	460	20	500,000
	480	20	500,000
	500	20	500,000
	520	20	500,000
	530	20	500,000
	550	20	500,000
	600	20	500,000
	630	20	500,000
	660	20	500,000
	813	4.0	2,500,000
	869	4.0	2,500,000
	1001	4.0	2,500,000
	1451	4.0	2,500,000
	1555	4.0	2,500,000
	2155	20	500,000
E	420	4.0	2,500,000
	440	4.0	2,500,000
	460	4.0	2,500,000
	480	4.0	2,500,000
	500	4.0	2,500,000
	520	4.0	2,500,000
	530	4.0	2,500,000
	550	4.0	2,500,000
	600	4.0	2,500,000
	630	4.0	2,500,000
	1289	2.0	5,000,000
	1451	4.0	2,500,000
	1555	2.0	5,000,000
